# Implementing user‐defined atlas‐based auto‐segmentation for a large multi‐centre organisation: the Australian Experience

**DOI:** 10.1002/jmrs.359

**Published:** 2019-10-28

**Authors:** Yunfei Hu, Mikel Byrne, Ben Archibald‐Heeren, Kenton Thompson, Andrew Fong, Marcel Knesl, Amy Teh, Eve Tiong, Richard Foster, Paul Melnyk, Michelle Burr, Amelia Thompson, Jiy Lim, Luke Moore, Fiona Gordon, Rylie Humble, Anna Hardy, Saul Williams

**Affiliations:** ^1^ ICON Cancer Centre Gosford Gosford New South Wales Australia; ^2^ Centre for Radiation Medical Physics University of Wollongong Wollongong New South Wales Australia; ^3^ ICON Cancer Centre Wahroonga Sydney Adventist Hospital Wahroonga New South Wales Australia; ^4^ Peter MacCallum Cancer Centre Melbourne Victoria Australia; ^5^ ICON Cancer Centre Maroochydore Maroochydore Queensland Australia; ^6^ Sydney Adventist Hospital Clinical School Sydney Medical School University of Sydney Sydney New South Wales Australia; ^7^ ICON Cancer Centre Midland Midland Western Australia Australia; ^8^ ICON Cancer Centre Hobart Hobart Tasmania Australia; ^9^ ICON Cancer Centre Cairns Liz Plummer Cancer Care Centre Cairns Queensland Australia; ^10^ ICON Cancer Centre Springfield Level 1, Cancer Care Centre Mater Private Hospital Springfield Queensland Australia; ^11^ ICON Cancer Centre Rockingham Rockingham Western Australia Australia

**Keywords:** Atlas‐based auto‐segmentation, contouring, efficiency gain, multi‐centre organisation, radiotherapy

## Abstract

**Introduction:**

Contouring has become an increasingly important aspect of radiation therapy due to inverse planning, and yet is extremely time‐consuming. To improve contouring efficiency and reduce potential inter‐observer variation, the atlas‐based auto‐segmentation (ABAS) function in Velocity was introduced to ICON cancer centres (ICC) throughout Australia as a solution for automatic contouring.

**Methods:**

This paper described the implementation process of the ABAS function and the construction of user‐defined atlas sets and compared the contouring efficiency before and after the introduction of ABAS.

**Results:**

The results indicate that the main limitation to the ABAS performance was Velocity's sub‐optimal atlas selection method. Three user‐defined atlas sets were constructed. Results suggested that the introduction of the ABAS saved at least 5 minutes of manual contouring time (*P *<* *0.05), although further verification was required due to limitations in the data collection method. The pilot rollout adopting a ‘champion’ approach was successful and provided an opportunity to improve the user‐defined atlases prior to the national implementation.

**Conclusion:**

The implementation of user‐defined ABAS for head and neck (H&N) and female thorax patients at ICCs was successful, which achieved at least 5 minutes of efficiency gain.

## Graphical abstract

To improve contouring efficiency and reduce potential inter‐observer variation, the atlas‐based auto‐segmentation (ABAS) function was introduced to ICON cancer centres (ICC) throughout Australia. Three user‐defined atlas sets were constructed, which saved at least 5 minutes of manual contouring time.

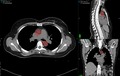

## Introduction

To fully exploit the advantages of inverse planning in radiation therapy, all target volumes and critical structures must be contoured before treatment planning. This time‐consuming process may be repeated multiple times during a treatment course because of tumour response or changes in patient weight or anatomy. When manual contouring is performed, large inter‐observer organ‐at‐risk (OAR) contouring variations have been reported, which may significantly affect dosimetric parameters. These differences impede the study of late side effects and establishment of a reliable normal tissue complication probability model.^1^, ^2^

One solution to this is atlas‐based auto‐segmentation (ABAS), a tool that automatically contours the OAR volumes. ABAS is the process of performing segmentation on a new image set using the knowledge of a prior segmentation that has had the structures of interest labelled.^3^. In addition to the benefit of reducing inter‐observer OAR contouring variations,^4^, ABAS has the potential to significantly reduce contouring time and improve planning efficiency.^5^ Multiple studies have reported that while manual contouring of the head and neck (H&N) and the breast areas can take anywhere between 18.6 min for delineating a CTV of the breast and 180 min for delineating multiple organs of the H&N, ABAS can reduce the contouring time up to 30–40%,^6^, ^7^, ^8^, ^9^ thereby lowering the contouring burden, allowing more normal tissues to be delineated and included in optimisation for intensity‐modulated radiation therapy to fully exploit known dose–volume effects.^7^.

ICON currently has 22 radiation therapy centres located across Australia. Implementing ABAS in a large multi‐centre organisation has the potential to provide the following major advantages: (1) increase contouring accuracy by reducing inter‐observer variations;^4^, ^5^, ^6^, ^7^, ^8^, ^9^, ^10^ (2) reduce contouring time and therefore improve planning efficiency;^4^, ^5^, ^6^, ^7^, ^8^, ^9^, ^10^ and (3) assist with implementing a uniform region‐of‐interest (ROI) naming convention, which will be beneficial for future automation implementation and data mining. However, implementation across a large number of centres also introduces a few challenges, mainly due to inconsistencies in (1) patient positioning techniques adopted at different centres; (2) image quality across different CT scanners; and (3) contouring guidelines followed by radiation oncologists (ROs) at different centres, all of which can degrade the performance of ABAS.

To fully utilise the benefits of auto‐segmentation while ensuring its safe and standardised implementation, a national project was undertaken to implement user‐defined ABAS that suited the clinical needs of ICON cancer centres (ICC). After analysing the patient profile of ICCs, it was concluded that implementing ABAS for H&N, female thorax and male pelvis patients was the most beneficial, as these types of patients constituted more than 80% of all patients. Velocity (version 4.0; Varian, Palo Alto, CA) software was utilised, whose image registration algorithms and ABAS functions have been validated by multiple studies.^9^, ^11^, ^12^, ^13^, ^14^

A number of previous studies have reported on the validation of ABAS for various systems,^3^, ^4^, ^6^, ^7^, ^8^, ^9^, ^15^ but few^10^, ^16^ have reported the implementation process of ABAS in a multi‐centre setting. This paper reports the procedures and findings of the ICON national ABAS implementation project, which included steps of data collection, user‐defined atlas construction, pilot rollout and preparation for national rollout, so as to provide reference for the implementation of ABAS in a large multi‐centre organisation.

## Methods

Statement: Ethics approval of this paper was exempt by the Research Office at Northern Sydney Local Health District (NSLHD).

### Atlas data collection

An expert panel, which constituted 6 ROs, 12 radiation therapists (RTs) and 3 physicists, was formed to implement the Velocity ABAS functions to all ICCs. Clinical data sets for H&N, female thorax and male pelvis patients treated at various ICC sites between January 2017 and March 2018 were retrospectively collected, including 48 H&N patients from 6 centres, 46 female thorax patients from 4 centres and 50 male pelvis patients from 6 centres. The panel then reviewed the image quality of these data sets and excluded 3 H&N patients, 5 breast patients and 6 prostate patients due to significant artefact or sub‐optimal image quality. During the review, it was noted that the collected patients varied in terms of body mass index (BMI), geometry (e.g. disease side, arm position and existence of breast implants in breast patients) and set up position (e.g. use of wing board vs. S board in breast patients). This variety could actually benefit the atlas database construction by improving its coverage of patient types. All collected patients were treated in the head‐first‐supine (HFS) position.

To improve the coverage and usefulness of the atlas, the expert panel proposed a structure list that should be included in an ideal atlas for each anatomical area, as shown in Table 1.

**Table 1:** List of structures to be included in the atlases.

H&N		Thorax (Female)		Pelvis (Male)
Hyoid bone	Larynx	Aortic arches	R3 axillary lymph nodes	Bowel bag
Left brachial plexus	Left lens	Descending aorta	L4E supraclavicular lymph nodes	Bladder
Right brachial plexus	Right lens	Pulmonary arteries	L4R supraclavicular lymph nodes	Pelvic bone
Brain	Lips	Left clavicle bone	R4E supraclavicular lymph nodes	Anal canal
Brainstem	Mandible	Right clavicle bone	R4R supraclavicular lymph nodes	External
Oral cavity	Medial constrict muscle	Left humeral head	Left lung	Left head of femur
Cerebellum	Oesophagus	Right humeral head	Right lung	Right head of femur
Cerebrum	Optic chiasm	Sternum	Left latissimus dorsi muscle	Left femur
Left cochlea	Left optic nerve	Left brachial plexus	Right latissimus dorsi muscle	Right femur
Right cochlea	Right optic nerve	Right brachial plexus	Left pectoralis major muscle	Penile bulb
Left lacrimal gland	Left parotid	Left breast	Right pectoralis major muscle	Prostate
Right lacrimal gland	Right parotid	Right breast	Oesophagus	Rectum
Left submandibular gland	Left retina	Bronchial tree	Ribs	Sacrum
Right submandibular gland	Right retina	Carina	Left scapula	Seminal vesicle
Left globe	Spinal cord	Chest wall	Right scapula	Proximal seminal vesicle
Right globe	Thyroid	Heart	Spinal column	Sigmoid
Left humerus	Tongue	Liver	Spinal cord	
Right humerus	Trachea	L1 axillary lymph nodes	Spleen	
Temporomandibular joint		L2 axillary lymph nodes	Trachea	
		L3 axillary lymph nodes	Inferior vena cava	
		R1 axillary lymph nodes	Superior vena cava	
		R2 axillary lymph nodes		

The collected data sets already included a certain number of contours from the previous treatment. RTs and physicists from the expert panel then reviewed these existing structures and modified them if necessary, as well as delineating those contours listed in Table 1 but were not included originally in the data set. The contours were reviewed and delineated following the Radiation Therapy Oncology Group (RTOG) contouring consensus.^17^ After the contours were complete, each data set was reviewed by an RO from the expert panel who specialised in the particular anatomical area, who would make changes to the contours if necessary. Due to limited staffing levels, by the end of the process, the number of patients reviewed out of all the collected patients was 29 H&N patients, 27 female thorax patients and 23 male pelvis patients. These reviewed patients were used to build the user‐defined atlases in the next stage. The contouring and review process took a total of 2 months.

### User‐defined atlas construction and assessment

Velocity 4.0 provides some tools to improve the performance of the atlases. The three major ones are as follows:^18^

- Utilising model‐based segmentation for individual structures. A small number of structures (brainstem, cerebellum, cerebrum, spinal cord, eyes, lungs and mandible) can be applied with a model‐based refinement already built in Velocity by adding a suffix ‘Refined’ to the end of the structure name.
- Utilising a local deformable registration B‐spline algorithm for individual structures. In Velocity, if a structure name has the suffix ‘Shaped’, a local deformable registration will be performed around the structure to obtain a better match prior to the creation of the structure. This feature can be applied to any structure.
- Exclusion Area for Atlas Sets. In Velocity, the best fit atlas set matching relies on matching bony anatomy of the atlas to a new planning volume. To improve the matching result, the vendor suggests that users create a structure to exclude high‐contrast artefact, such as couch, arms, dental artefact or contrast‐enhancing agent.

**Figure 1:**
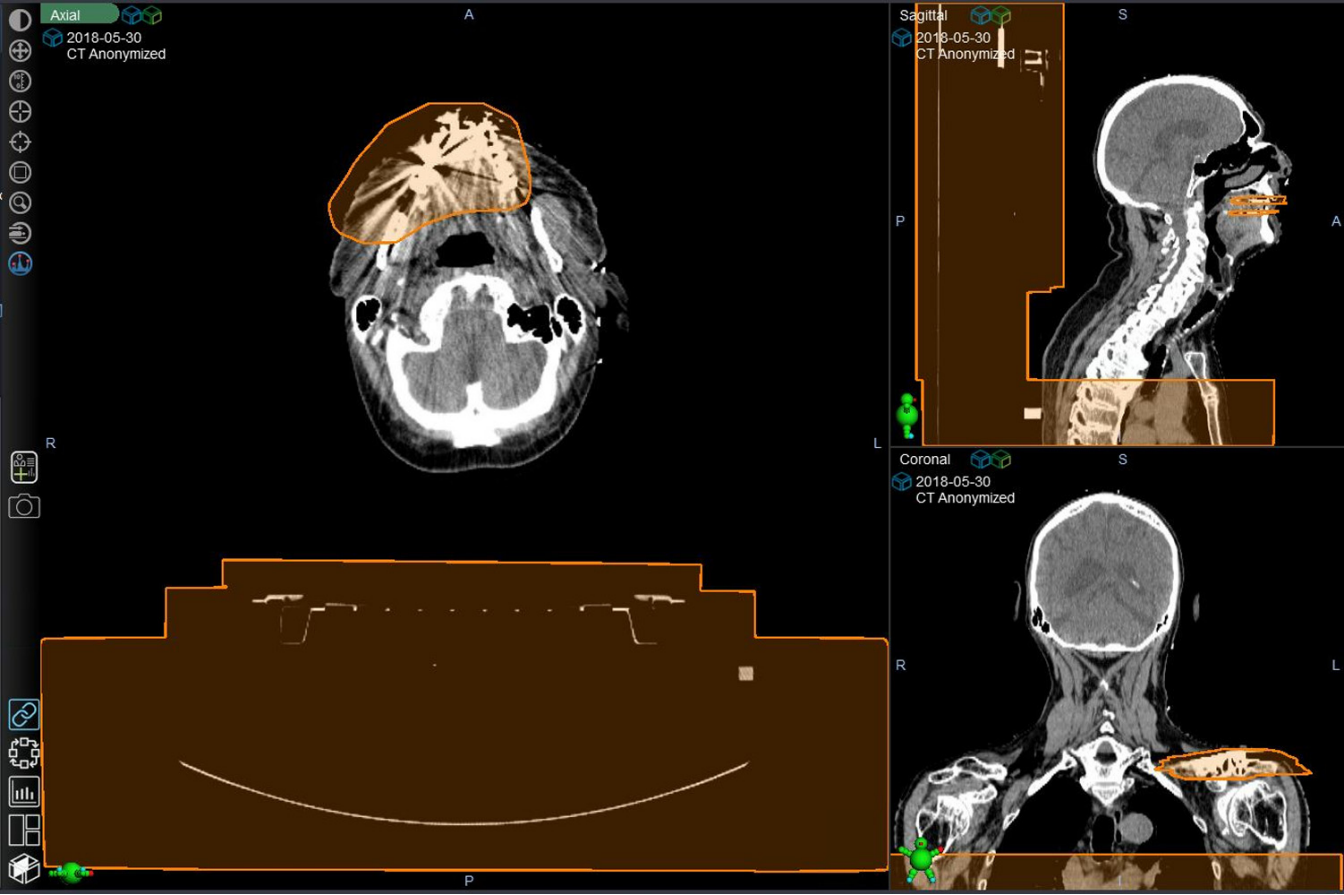
Example of the use of ‘Exclusion Area for Atlas Sets’ to exclude high‐density materials and anatomy beyond the ROI.

The Exclusion Area for Atlas Sets feature in Velocity was utilised to exclude high‐density materials and regions beyond the region of interest (ROI) in the CT data sets. An example of the exclusion area is shown in Figure 1 (shown in *Orange*), where the H&N patient's dental implant (and its artefact), couch and inferior part of the scan were included in the exclusion area, so that when system searches for the best match, it focuses on the region where most contours are located while minimising the influence of artefact from high‐contrast materials.

The next stage was to independently test the efficacy of the Refined and Shaped tools on structure delineation. For this purpose, 3 test atlases were created for each area (2 for male pelvis, as no structures in the area can be refined in Velocity), which were Default Atlas Set, where no corrections were used for all structures; Refined Atlas Set, where structures that could be refined were all added with a ‘Refined’ suffix to apply a model refined to the deformation; and Shaped Atlas Set, where all structures were added with a ‘Shaped’ suffix to perform a local deformation. Ten data sets were then randomly selected from the reviewed patient cohort to generate the test atlases, and another 5 data sets from the reviewed patient cohort were selected, where the test atlases were run and the performances were compared.

In this study, the main quantitative indicator of contour agreement adopted was the dice similarity coefficient (DSC);^19^ DSC = $\frac{2|V_{\mathrm{seg}}\cap V_{\mathrm{man}}|}{|V_{\mathrm{seg}}+V_{\mathrm{man}}|}$, where Vseg and Vman denote the volume of the ABAS contour and that of the manual contour, respectively.^20^ The DSC of two selected structures was calculated by Velocity. DSC approaches 1.0 when two structures overlap exactly. One study has recommended a DSC of 0.7 to be considered a good overlap,^21^ whereas others have instead suggested 0.8.^22^, ^23^. However, DSC assigns double value to the overlap area and its interpretation as concordance measure can provide false impression of high agreement.^23^. In addition, it over‐penalises small structures but is too permissive for large structures.^24^ Therefore, in addition to DSC, the expert panel also performed a qualitative assessment of the agreement between the automatically contoured and the manually contoured structures by visual inspection, which is the same approach adopted in a previous study.^25^ In addition, the panel has introduced the concept of ‘structure utility’ as a subjective assessment of the utility of including a structure in the atlas, taking into account its frequency of use in planning and ease of contouring manually. In this study, the structure utility has three levels: ‘High’, ‘Moderate’ and ‘Low’; structures that are more frequently used and more difficult to manually contour have a higher structure utility. Based on the results, the panel then decided whether a structure should be included in the atlas, and if included, how it should be modelled (Default, Refined or Shaped). Finally, all reviewed patients, including 29 H&N patients, 27 female thorax patients and 23 male pelvis patients, were added to the final atlases to maximise the number of cases in these atlases.

### Efficiency gain estimation and user feedback collection

Prior to implementation, a baseline study was conducted to record the average time an RT spent on contouring. Three ICC centres were selected, and the RTs of the selected centres were required to record their contouring time by creating a ‘Contouring’ task in the patient information system (Aria version 15.1; Varian, Palo Alto, CA) when they started contouring and completed the task when they finished contouring. The median time recorded using this method was established as the baseline of the efficiency gain estimation. However, the data points collected this way were not stratified between anatomical sites or plan types. A total of 387 data points were collected using this method.

When the atlases were constructed, they were first piloted at the 3 ICC centres where the baseline data were collected. An RT from each centre was specifically trained to act as the local expert, known as the ‘RT champion’. For each patient where an atlas was performed, the RT champion would review and score the contours generated by the atlas on a scale of ‘No Change’ (the structure requires no editing), ‘Minor Change’ (less than 10% of the structure required editing), ‘Major Change’ (less than 50% of the structure required editing) or ‘Delete and Restart’ (which means the outcome was not usable). Additionally, the ‘champion’ RTs were required to record the total time spent on running the atlas and then editing the contours until satisfactory in the feedback form. Due to limited duration of the piloting phase, only 20 data points were collected. These 20 data points were then compared to the 387 baseline points, the *P* value of which was calculated by the one‐way ANOVA test. The result was significant if *P *<* *0.05.

Other basic system function tests, such as volume fidelity, system integrity, atlas generation reproducibility and end‐to‐end tests, were performed prior to the implementation. The expert panel also created a naming script that converts the atlas structure names to standard naming conventions following the recommendation of TG‐263,^26^ which greatly facilitates all future automation and scripting projects, as well as potential big data mining.

## Results and Discussions

### Atlas construction

**Figure 2:**
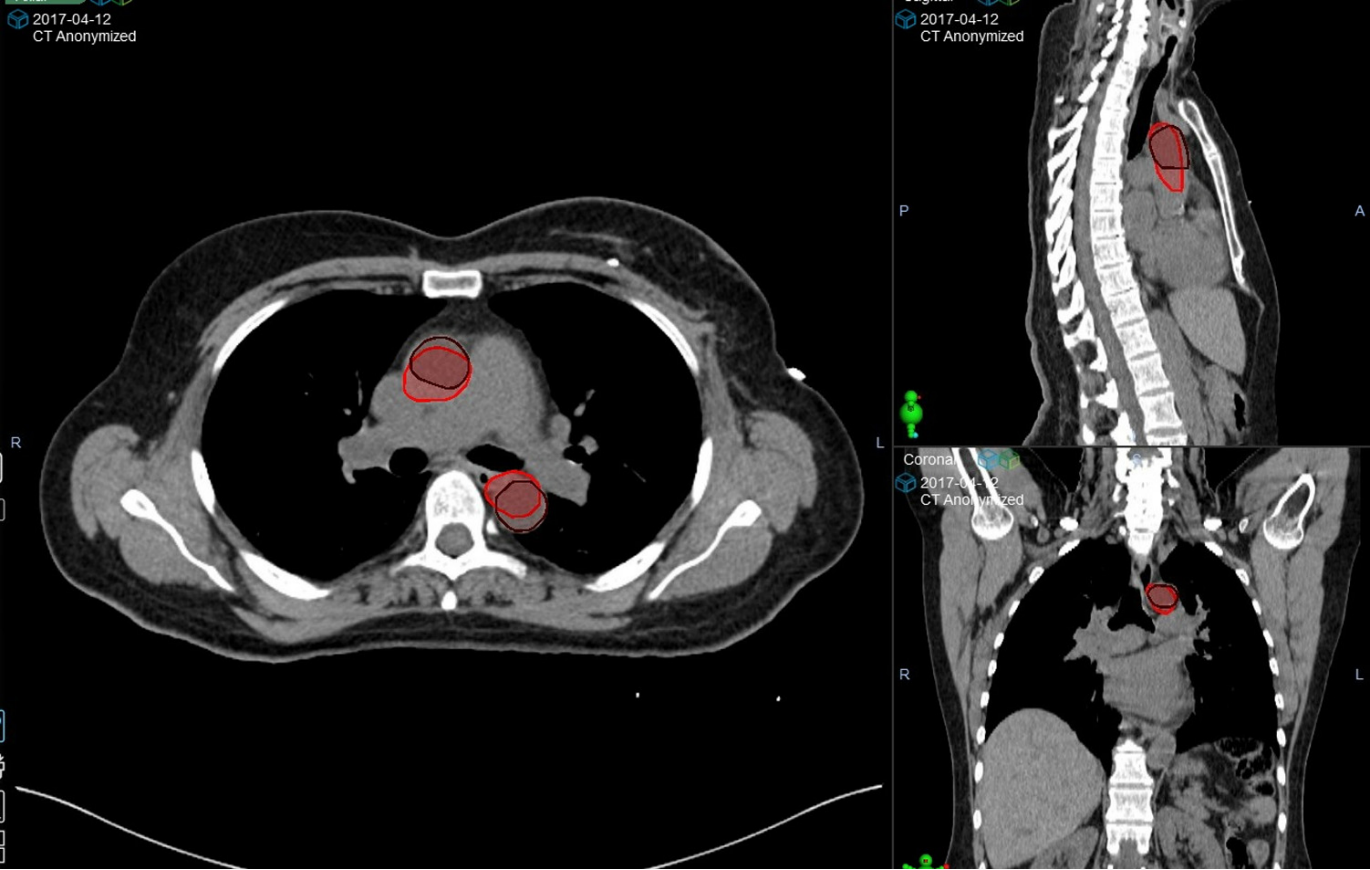
Visual inspection agreement of the manual aortic arch contour (brown) and the automatic aortic arch contour (red). Although the DSC was relatively high (0.802), the two contours’ size, location and extension were significantly different. Combined with its low structure utility, this structure was excluded from the final atlas set.

Tables 2, 3, 4 list the inclusion/exclusion of structures based on 3 factors: DSC, visual inspection agreement and structure utility. A structure was included in the final atlas if (1) DSC > 0.7,^21^ the visual assessment result was ‘Moderate’ to ‘Good’ and the structure utility was ‘Medium’ to ‘High’; or (2) DSC < 0.7, but the visual assessment result was ‘Moderate’ to ‘Good’ and the structure utility was ‘High’. The reason why visual assessment was adopted in combination with DSC as the selection criteria was that DSC over‐penalises small structures but is too permissive for large structures.^24^ An example of a structure (aortic arches) whose DSC was high (0.802) but was excluded from the atlas due to a poor visual agreement and a low structure utility is given in Figure 2. Structures that were deemed acceptable for inclusion in the final atlas, as well as the final contour propagation modality selected (Default, Shaped or Refined), are indicated in bold in Tables 2, 3, 4. For structures that are not shown in bold in any of the columns, this indicates that none of the atlases were deemed acceptable, and these structures were correspondingly excluded from the final atlas.

**Table 2:** Structure inclusion/exclusion of the H&N atlas set. Structures that were included in the final atlas are indicated in bold.

Structure name	Structure utility	Default		Shaped		Refined	
		Average DSC (range of DSC)	Visual assessment	Average DSC (range of DSC)	Visual assessment	Average DSC (range of DSC)	Visual assessment
Hyoid bone	Low	0.121 (0.052 – 0.173)	Poor	0.438 (0.000 – 0.750)	Poor	N/A	N/A
Left brachial plexus	High	0.103 (0.072 – 0.152)	Poor	0.153 (0.039 – 0.292)	Poor	N/A	N/A
Right brachial plexus	High	0.093 (0.007 – 0.202)	Poor	0.162 (0.060 – 0.306)	Poor	N/A	N/A
**Brain**	High	0.962 (0.959 – 0.964)	Good	**0.973 (0.968 – 0.977)**	**Good**	N/A	N/A
**Brainstem**	High	0.678 (0.590 – 0.755)	Moderate	**0.707 (0.594 – 0.785)**	**Moderate**	0.678 (0.591 – 0.755)	Moderate
**Oral cavity**	Medium	0.702 (0.646 – 0.774)	Good	**0.734 (0.684 – 0.803)**	**Good**	N/A	N/A
Cerebellum	Low	0.627 (0.476 – 0.800)	Poor	0.640 (0.544 – 0.823)	Poor	0.631 (0.476 – 0.808)	Poor
Cerebrum	Low	0.913 (0.912 – 0.915)	Good	0.911 (0.907 – 0.918)	Good	0.920 (0.917 – 0.926)	Good
Left cochlea	High	0.000 (0.000 – 0.000)	Poor	0.000 (0.000 – 0.000)	Poor	N/A	N/A
Right cochlea	High	0.177 (0.000 – 0.532)	Poor	0.000 (0.000 – 0.000)	Poor	N/A	N/A
**Left lacrimal gland**	High	0.142 (0.000 – 0.301)	Moderate	**0.322 (0.209 – 0.513)**	**Moderate**	N/A	N/A
**Right lacrimal gland**	High	0.123 (0.099 – 0.157)	Moderate	**0.094 (0.027 – 0.146)**	**Moderate**	N/A	N/A
Left submandibular gland	Low	0.469 (0.344 – 0.537)	Poor	0.530 (0.475 – 0.558)	Poor	N/A	N/A
Right submandibular gland	Low	0.373 (0.220 – 0.478)	Poor	0.361 (0.287 – 0.449)	Poor	N/A	N/A
**Left globe**	High	0.752 (0.644 – 0.862)	Moderate	0.852 (0.790 – 0.901)	Good	**0.870 (0.799 – 0.908)**	**Good**
**Right globe**	High	0.762 (0.703 – 0.804)	Moderate	0.846 (0.782 – 0.896)	Good	**0.841 (0.780 – 0.915)**	**Good**
Left humerus	Low	0.701 (0.541 – 0.929)	Poor	0.850 (0.754 – 0.943)	Moderate	N/A	N/A
Right humerus	Low	0.754 (0.592 – 0.897)	Poor	0.880 (0.781 – 0.958)	Moderate	N/A	N/A
Temporomandibular joint	Medium	0.422 (0.388 – 0.479)	Poor	0.597 (0.472 – 0.780)	Poor	N/A	N/A
Larynx	Medium	0.347 (0.239 – 0.503)	Poor	0.364 (0.142 – 0.569)	Poor	N/A	N/A
Left lens	High	0.355 (0.253 – 0.460)	Poor	0.426 (0.066 – 0.644)	Poor	N/A	N/A
Right lens	High	0.308 (0.046 – 0.538)	Poor	0.270 (0.000 – 0.471)	Poor	N/A	N/A
Lips	Low	0.427 (0.206 – 0.544)	Poor	0.497 (0.371 – 0.620)	Poor	N/A	N/A
**Mandible**	High	0.635 (0.510 – 0.719)	Poor	0.727 (0.642 – 0.836)	Moderate	**0.868 (0.791 – 0.924)**	**Good**
Constrict muscle	High	0.264 (0.119 – 0.395)	Poor	0.407 (0.366 – 0.472)	Poor	N/A	N/A
Oesophagus	Medium	0.410 (0.330 – 0.457)	Poor	0.431 (0.311 – 0.519)	Poor	N/A	N/A
**Optic chiasm**	High	0.308 (0.157 – 0.399)	Poor	**0.393 (0.256 – 0.462)**	**Moderate**	N/A	N/A
**Left optic nerve**	High	0.302 (0.259 – 0.338)	Poor	**0.404 (0.063 – 0.661)**	**Moderate**	N/A	N/A
**Right optic nerve**	High	0.272 (0.151 – 0.459)	Poor	**0.520 (0.362 – 0.699)**	**Moderate**	N/A	N/A
**Left parotid**	High	**0.572 (0.399 – 0.715)**	**Moderate**	0.496 (0.430 – 0.615)	Moderate	N/A	N/A
**Right parotid**	High	**0.481 (0.160 – 0.702)**	**Moderate**	0.442 (0.174 – 0.666)	Poor	N/A	N/A
Left retina	Medium	0.045 (0.000 – 0.077)	Poor	0.205 (0.028 – 0.547)	Poor	N/A	N/A
Right retina	Medium	0.114 (0.088 – 0.131)	Poor	0.269 (0.068 – 0.472)	Poor	N/A	N/A
**Spinal cord**	High	0.574 (0.483 – 0.624)	Moderate	0.555 (0.431 – 0.651)	Moderate	**0.619 (0.379 – 0.846)**	**Good**
Thyroid	Medium	0.330 (0.151 – 0.566)	Poor	0.356 (0.160 – 0.667)	Poor	N/A	N/A
Tongue	Low	0.313 (0.000 – 0.532)	Poor	0.238 (0.000 – 0.648)	Poor	N/A	N/A
**Trachea**	High	0.620 (0.551 – 0.690)	Moderate	**0.677 (0.586 – 0.732)**	**Moderate**	N/A	N/A

**Table 3:** Structure inclusion/exclusion of the female thorax and thorax nodes atlas set. Structures that were included in the final atlas are indicated in bold.

Structure name	Structure utility	Default		Shaped		Refined	
		Average DSC (range of DSC)	Visual assessment	Average DSC (range of DSC)	Visual assessment	Average DSC (range of DSC)	Visual assessment
Aortic arches	Low	0.650 (0.641 – 0.662)	Poor	0.733 (0.688 – 0.778)	Poor	N/A	N/A
Descending aorta	Low	0.669 (0.527 – 0.685)	Poor	0.813 (0.676 – 0.814)	Poor	N/A	N/A
Pulmonary arteries	Low	0.644 (0.625 – 0.662)	Poor	0.695 (0.651 – 0.738)	Poor	N/A	N/A
**Airways (combination of bronchial trees, carina and trachea)**	High	0.631 (0.524 – 0.706)	Poor	**0.716 (0.697 – 0.802)**	**Moderate**	N/A	N/A
Left clavicle bone	Low	0.552 (0.436 – 0.668)	Moderate	0.810 (0.746 – 0.873)	Moderate	N/A	N/A
Right clavicle bone	Low	0.578 (0.530 – 0.626)	Poor	0.777 (0.719 – 0.834)	Poor	N/A	N/A
Left humeral head	Low	0.788 (0.747 – 0.829)	Moderate	0.846 (0.831 – 0.860)	Moderate	N/A	N/A
Right humeral head	Low	0.810 (0.780 – 0.840)	Moderate	0.850 (0.827 – 0.873)	Moderate	N/A	N/A
Sternum	Low	0.678 (0.605 – 0.726)	Poor	0.735 (0.666 – 0.804)	Poor	N/A	N/A
**Left brachial plexus**	High	**0.262 (0.163 – 0.361)**	**Moderate**	0.257 (0.140 – 0.428)	Poor	N/A	N/A
**Right brachial plexus**	High	**0.324 (0.067 – 0.341)**	**Moderate**	0.277 (0.128 – 0.367)	Moderate	N/A	N/A
**Left breast**	High	0.886 (0.703 – 0.895)	**Good**	**0.901 (0.839 – 0.921)**	Good	N/A	N/A
**Right breast**	High	0.723 (0.586 – 0.901)	**Moderate**	**0.791 (0.724 – 0.921)**	Good	N/A	N/A
Chest wall	Medium	0.726 (0.279 – 0.773)	Poor	0.740 (0.624 – 0.775)	Moderate	N/A	N/A
**Heart**	High	0.917 (0.793 – 0.924)	**Good**	**0.930 (0.911 – 0.935)**	Good	N/A	N/A
Liver	Low	0.864 (0.710 – 0.890)	Moderate	0.926 (0.841 – 0.934)	Good	N/A	N/A
**L1 axillary lymph nodes**	High	**0.656 (0.308 – 0.675)**	**Moderate**	0.579 (0.476 – 0.682)	Moderate	N/A	N/A
**L2 axillary lymph nodes**	High	**0.638 (0.402 – 0.680)**	**Moderate**	0.507 (0.459 – 0.684)	Moderate	N/A	N/A
**L3 axillary lymph nodes**	High	0.536 (0.256 – 0.564)	Moderate	**0.542 (0.436 – 0.585)**	**Moderate**	N/A	N/A
**R1 axillary lymph nodes**	High	**0.706 (0.247 – 0.825)**	**Moderate**	0.637 (0.488 – 0.785)	Moderate	N/A	N/A
**R2 axillary lymph nodes**	High	**0.729 (0.093 – 0.809)**	**Moderate**	0.673 (0.544 – 0.752)	Moderate	N/A	N/A
**R3 axillary lymph nodes**	High	**0.682 (0.469 – 0.716)**	**Moderate**	0.657 (0.500 – 0.696)	Moderate	N/A	N/A
**L4E supraclavicular lymph nodes**	High	**0.654 (0.368 – 0.684)**	**Moderate**	0.620 (0.528 – 0.655)	Moderate	N/A	N/A
**L4R supraclavicular lymph nodes**	High	0.650 (0.464 – 0.651)	Moderate	**0.655 (0.610 – 0.699)**	**Moderate**	N/A	N/A
**R4E supraclavicular lymph nodes**	High	**0.635 (0.284 – 0.694)**	**Moderate**	0.492 (0.470 – 0.526)	Moderate	N/A	N/A
**R4R supraclavicular lymph nodes**	High	**0.692 (0.490 – 0.716)**	**Moderate**	0.590 (0.510 – 0.608)	Moderate	N/A	N/A
**Left lung**	High	0.936 (0.773 – 0.929)	Moderate	0.960 (0.947 – 0.966)	Good	**0.975 (0.884 – 0.978)**	**Good**
**Right lung**	High	0.952 (0.820 – 0.954)	Moderate	0.967 (0.960 – 0.970)	Good	**0.974 (0.947 – 0.978)**	**Good**
**Left latissimus dorsi muscle**	Medium	0.684 (0.128 – 0.716)	**Moderate**	**0.795 (0.535 – 0.830)**	Good	N/A	N/A
**Right latissimus dorsi muscle**	Medium	0.612 (0.237 – 0.687)	**Moderate**	**0.786 (0.605 – 0.802)**	Good	N/A	N/A
**Left pectoralis major muscle**	Medium	0.667 (0.257 – 0.668)	**Moderate**	**0.750 (0.516 – 0.771)**	Moderate	N/A	N/A
**Right pectoralis major muscle**	Medium	0.600 (0.037 – 0.657)	**Moderate**	**0.721 (0.612 – 0.735)**	Moderate	N/A	N/A
Oesophagus	Medium	0.516 (0.405 – 0.547)	Poor	0.591 (0.566 – 0.642)	Poor	N/A	N/A
Ribs	Medium	0.465 (0.129 – 0.508)	Poor	0.573 (0.376 – 0.587)	Poor	N/A	N/A
Left scapula	Low	0.556 (0.482 – 0.629)	Moderate	0.673 (0.582 – 0.764)	Poor	N/A	N/A
Right scapula	Low	0.496 (0.404 – 0.587)	Poor	0.652 (0.557 – 0.747)	Poor	N/A	N/A
Spinal column	Low	0.744 (0.738 – 0.750)	Poor	0.802 (0.758 – 0.845)	Moderate	N/A	N/A
**Spinal cord**	High	0.648 (0.636 – 0.689)	Good	0.717 (0.665 – 0.825)	Good	**0.739 (0.659 – 0.833)**	**Good**
Spleen	Low	0.659 (0.514 – 0.687)	Moderate	0.829 (0.687 – 0.848)	Moderate	N/A	N/A
Inferior vena cava	Low	0.414 (0.179 – 0.594)	Poor	0.365 (0.103 – 0.453)	Poor	N/A	N/A
Superior vena cava	Low	0.572 (0.470 – 0.673)	Poor	0.603 (0.545 – 0.661)	Poor	N/A	N/A

**Table 4:** Structure inclusion/exclusion of the male pelvis atlas set. Structures that were included in the final atlas are indicated in bold.

Structure name	Structure utility	Default		Shaped		Refined	
		Average DSC (range of DSC)	Visual assessment	Average DSC (range of DSC)	Visual assessment	Average DSC (range of DSC)	Visual assessment
Bowel bag	Medium	0.660 (0.491 – 0.66)	Poor	0.658 (0.477 – 0.899)	Poor	N/A	N/A
Bladder	High	0.723 (0.659 – 0.764)	Poor	0.809 (0.793 – 0.820)	Moderate	N/A	N/A
Pelvic bone	Low	0.873 (0.852 – 0.884)	Moderate	0.910 (0.894 – 0.921)	Moderate	N/A	N/A
Anal canal	Medium	0.275 (0.075 – 0.487)	Poor	0.433 (0.200 – 0.672)	Poor	N/A	N/A
Left head of femur	High	0.864 (0.833 – 0.908)	Moderate	0.893 (0.874 – 0.919)	Moderate	N/A	N/A
Right head of femur	High	0.846 (0.843 – 0.916)	Moderate	0.903 (0.874 – 0.941)	Good	N/A	N/A
Left femur	Medium	0.535 (0.180 – 0.933)	Poor	0.608 (0.378 – 0.954)	Moderate	N/A	N/A
Right femur	Medium	0.511 (0.127 – 0.925)	Poor	0.602 (0.387 – 0.946)	Moderate	N/A	N/A
Penile bulb	High	0.411 (0.151 – 0.563)	Poor	0.456 (0.067 – 0.687)	Poor	N/A	N/A
Prostate	High	0.483 (0.436 – 0.530)	Poor	0.589 (0.558 – 0.620)	Poor	N/A	N/A
Rectum	High	0.395 (0.281 – 0.483)	Poor	0.615 (0.534 – 0.709)	Poor	N/A	N/A
Sacrum	Low	0.847 (0.782 – 0.894)	Moderate	0.891 (0.846 – 0.927)	Moderate	N/A	N/A
Seminal vesicle	High	0.079 (0.010 – 0.147)	Poor	0.226 (0.050 – 0.401)	Poor	N/A	N/A
Proximal seminal vesicle	Low	0.146 (0.127 – 0.161)	Poor	0.441 (0.352 – 0.542)	Poor	N/A	N/A
Sigmoid	Medium	0.057 (0.024 – 0.079)	Poor	0.083 (0.014 – 0.119)	Poor	N/A	N/A

From the DSC results, it is observed that (1) in general, the DSCs are relatively low, with almost half of the structures (44/93, 47.3%) having a DSC < 0.6, which demonstrates the limitation of the ABAS; (2) for most structures, performing the local deformation registration (Shaped) improves the agreement, consistent with previous literature.^27^. However, it is noted that in cases where the atlas selected closely matches the patient's anatomy, soft‐tissue structures that are closely related to surrounding bone structures (e.g. nodes) deliver better results under the Default setting; (3) for those structures that are enabled of model refinement (Refined), the refined structure generally has a higher DSC (an average of 0.06 escalation compared to Default and 0.02 escalation compared to Shaped) than that of the other two modalities; (4) bone structures generally have a better DSC than soft‐tissue structures. This is because in Velocity, a similarity matrix based on the bone geometry is used to calculate the similarity of the atlas CT and the new CT. This matrix does not consider any soft‐tissue characteristics. Therefore, in most cases the soft‐tissue matching between the atlas CT and the new CT is worse than the bone matching, thereby resulting in a poorer ABAS outcome for soft‐tissue structures.

**Figure 3:**
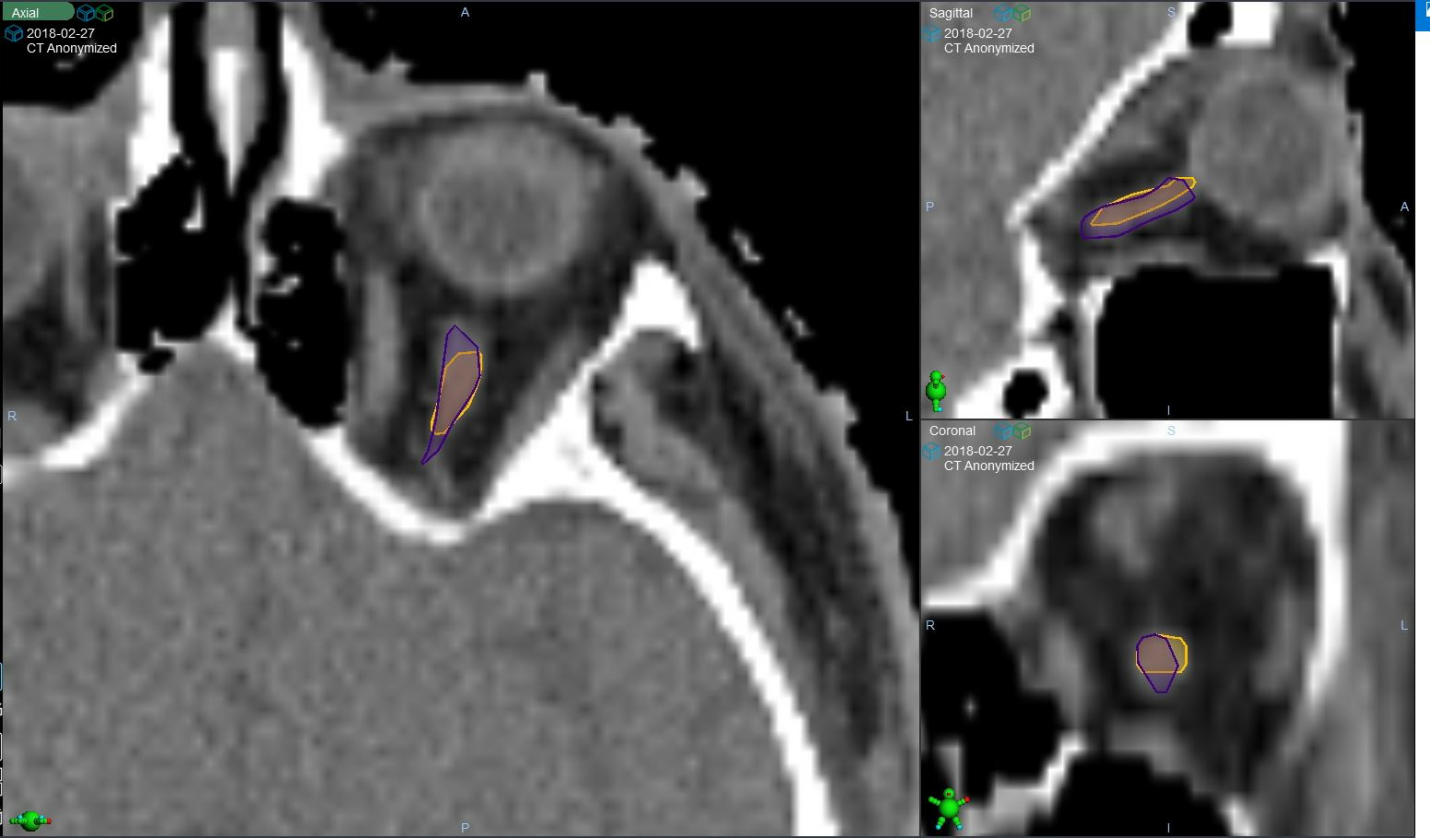
Visual inspection agreement of the manual left optic nerve contour (orange) and the automatic left optic nerve contour (purple). Although the DSC was low (0.452), the two contours’ size, location and extension were close. Therefore, this structure was included in the final atlas set despite the low DSC.

It is noted that the H&N atlas provides the best outcome compared to the female thorax and the male pelvis atlases, which is consistent with previous studies.^3^, ^9^ In particular, structures with clear boundaries, such as brain, mandible and spinal cord, all demonstrate a DSC of above 0.9. Structures with smaller volumes tend to show lower DSCs due to the nature of the definition, but visual inspection indicates that although some of these structures’ DSCs are low, their visual alignments are acceptable, and auto‐segmentation provides a good estimation of where the structure is. Therefore, despite the low DSC scores, some small‐volume structures with high structure utilities are still included in the atlas set. An example (left optic nerve) is shown in Figure 3.

For the female thorax/thorax nodes atlas, the expert panel decided to split it into 2 sub‐atlases: one for organs and one for lymph nodes and muscles. The reason behind this is that (1) lymph nodes and muscles are less frequently required clinically. Therefore, including them in a single atlas set will result in unnecessary extension in the running time (by approximately 5 min), and after ABAS, users need to delete these structures that are not required clinically. Alternatively, keeping the nodes and muscles in a separate atlas avoids this problem; and (2) by splitting the atlas, the authors were able to use the ‘Exclusion Area’ function in Velocity to further limit the ROI for best‐matching atlas selection in the lymph node‐muscle atlas, so that Velocity focuses on the lymph node/muscle region when selecting a best‐matching atlas.

The atlas test results in the pelvic region are extremely poor. Among all the structures, only bony structures, such as the left and right femurs, generated an outcome that was clinically acceptable, but the structure utilities of these structures were low. As discussed above, this is due to the bony matrix adopted by Velocity when selecting the best‐matching atlas. While this particular matrix works well on H&N patients (which have multiple bony structures and well‐defined structure boundaries), it does not perform as well in the pelvic area, as important soft‐tissue features and variations in this area are ignored by the system, often causing an atlas CT with totally different organ geometries to be selected for the new CT. Additionally, there is no clear contrast between the critical structures in this area, such as between bladder and prostate, which further reduces the accuracy of deformable registration. Therefore, although previous studies suggested that some structures that were automatically contoured in Velocity could be used clinically after manual review and editing,^28^, ^29^ in this study the expert panel has decided that the current ABAS performance in the pelvic area does not support the establishment of a user‐defined atlas.

In summary, a total of 3 atlases, 1 for H&N and 2 for female thorax (1 for organ and 1 for nodes), were validated for clinical use. A total of 29 H&N patient data sets were included in the H&N atlas, and 27 female thorax patient data sets were included in each of the two female thorax atlases. The final structure list of all atlases is shown in Table 5.

**Table 5:** Final structure list of the 3 customer‐built atlases.

H&N atlas set		Female thorax atlas set	Thorax nodes atlas set	
Brain	Right lacrimal gland	Airways	Left brachial plexus	L4E supraclavicular lymph nodes
Brainstem	Mandible	Left breast	Right brachial plexus	L4R supraclavicular lymph nodes
Oral cavity	Optic chiasm	Right breast	External	R4E supraclavicular lymph nodes
External	Left optic nerve	External	L1 axillary lymph nodes	R4R supraclavicular lymph nodes
Left globe	Right optic nerve	Heart	L2 axillary lymph nodes	Left latissimus dorsi muscle
Right globe	Spinal cord	Left lung	L3 axillary lymph nodes	Right latissimus dorsi muscle
Left lacrimal gland	Trachea	Right lung	R1 axillary lymph nodes	Left pectoralis major muscle
		Spinal cord	R2 axillary lymph nodes	Right pectoralis major muscle
			R3 axillary lymph nodes	

### Efficiency gain estimation

**Figure 4:**
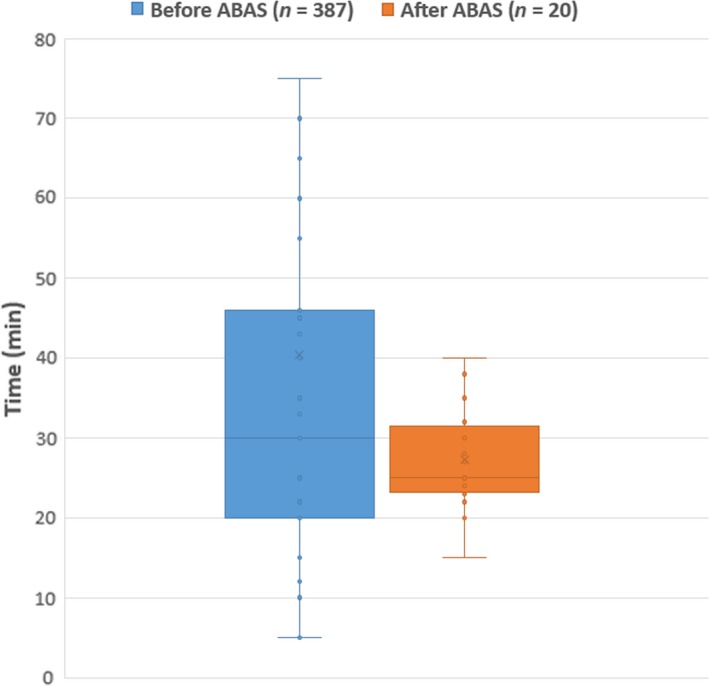
Boxplots of times RTs spent on contouring before and after the introduction of ABAS. *P *=* *0.0487 < 0.05, indicating that the difference is statistically significant.

Figure 4 shows the boxplots of times RTs spent on contouring before and after ABAS was implemented.

From Figure 4, it is observed that before ABAS was introduced, RTs spent approximately 20–46 min in contouring, with a median value of approximately 30 min. After ABAS was introduced, RTs now spend between 24 and 32 min on contouring including running the atlas and performing the necessary editing and post‐processing, with a median value of around 25 min. Out of this 25 min, an average of 15 min was spent on running the atlases, 5 min of which required user interaction. One‐way ANOVA test showed that the *P* value was 0.0478, indicating that the difference was statistically significant as *P *<* *0.05. However, it is worth mentioning that the compositions of the two data sets were substantially different. As was previously mentioned, the data points collected prior to the introduction of ABAS were not stratified between anatomical sites or plan types, whereas those collected afterwards only included breast and H&N patients. Therefore, the two groups were not directly comparable to conclude an apparent time difference. In addition, the expert panel believes that due to limitations in the data collection method, the baseline result underestimates the contouring time for H&N and female thorax patients, because (1) during data collection, it was not possible for the expert panel to identify the plan type and the treated anatomical area. Therefore, these data include the contouring times of electron plans and palliative plans, whose number of required contours is likely substantially smaller than that of curative inverse‐planned photon plans for H&N and female thorax patients; and (2) as mentioned in Section ‘Atlas data collection’, in most clinical cases, only the minimal number of structures was contoured. Structures that were considered not an organ of interest would not have been contoured due to the heavy workload.

In summary, although our results indicate that the implementation of ABAS leads to a 5‐minute (16.7%) reduction in the median contouring time, further analysis is required to verify this result. Due to the data collection method, the contouring time recorded prior to the introduction of ABAS underestimates the contouring time for H&N and female thorax patients, and consequently, this time saving can be potentially larger.

### User feedback collection

A pilot rollout at 3 ICCs was conducted using the aforementioned ‘RT champion’ approach prior to the national implementation. After ABAS, the corresponding planning RT reviews and adjusts any contours requiring editing and fills in the feedback form. The advantages of adopting the champion approach include the following: (1) it reduces the resources required to train RTs, while allowing a more personalised and in‐depth training provided to the individual; (2) it simplifies the communication chain and makes it easier for the expert panel to collect feedback; and (3) it introduces fewer interruptions to clinical workflow, making it easier for staff to accept change. The pilot rollout lasted 2 months at 3 centres, and the summarised feedback results are shown below in Table 6.

**Table 6:** Feedback statistics of all atlas structures used clinically.

Atlas set	No Change	Minor Change	Moderate Change	Delete and Restart
H&N (*n *=* *27)	19.3%	44.8%	18.7%	17.2%
Female thorax (*n *=* *37)	19.0%	44.8%	21.6%	14.6%

From the user's feedback, it is noted that for both anatomical areas, approximately 64% (64.1% for H&N and 63.8% for female thorax) of the ABAS structures were reported to require either no or minor change, which is considered an acceptable outcome. In the H&N atlas, the main structure that consistently needs to be deleted and restarted is the left and right brachial plexus, which accounts for 30% of that group. In the female thorax atlas, the main contributor to the ‘Delete and Restart’ group is left and right lung. This is because, although the DSC values of the lungs were high during testing (above 0.9), it was not perfect and still required a certain amount of manual editing. However, almost all planning systems had a threshold‐based lung contouring tool that could automatically delineate the lungs, the results of which required substantially less editing compared to those of Velocity. Therefore, most RTs chose to delete the lungs contoured by Velocity and instead use the threshold‐based tool in the planning system rather than editing it, resulting in the high ‘Delete and Restart’ rate of the structure. Based on the feedback, right and left brachial plexus, cerebellum, cerebrum and left and right humerus in the H&N atlas set, and sternum in the female thorax atlas set, were removed, as RTs tended to delete and restart these structures among most cases.

### Study limitations and future outlook

First, compared with previous studies,^3^, ^4^, ^5^, ^6^, ^7^, ^8^, ^13^ the number of ROs who participated in the review of contours was significantly smaller, which meant that inter‐observer variations were not well accounted for when building the database.

Second, there are some limitations with the collection of the efficiency data: (1) the baseline data points were not stratified between anatomical areas (e.g. H&N, female thorax or male pelvis) and plan types (e.g. radical vs. palliative, photon vs. electron), making the two data sets not directly comparable; and (2) baseline contouring differences among the RT champions from the 3 participating centres were not established, which could lead to a bias in the final result.

The authors have identified possible measures to further improve the efficiency and performance of ABAS, which include the following: automated contour editing and post‐processing via scripting; statistical based atlas selection to improve best‐matching atlas selection; subdivision of atlases based on patient cohort with increased patient data available; and automated atlas selection and atlas running upon CT import. Investigations in implementing these measures are currently underway and will be reported once the results are available.

## Conclusion

The ABAS function in Velocity was implemented to reduce the contouring time and to improve the output consistency. A total of 3 atlases were constructed for H&N and female thorax patients. A major limitation to the performance of the ABAS was Velocity's sub‐optimal atlas selection method, which adopts a bony matrix that ignores soft‐tissue features. Although it provided acceptable results in the H&N and female thorax areas, its performance in the pelvic region was not acceptable, and consequently, the authors did not create a user‐defined pelvis atlas set.

Although the efficiency study revealed that implementing ABAS on average saved 5 min of contouring time, further verification was required on this result due to limitations in the data collection method. A pilot rollout using a ‘champion’ approach provided valuable feedback and an opportunity for authors to improve the user‐defined atlases prior to the national implementation.
